# Malnutrition among students with visual impairment studying in integrated public schools of Nepal

**DOI:** 10.1017/jns.2025.10025

**Published:** 2025-08-06

**Authors:** Bijay Khatri, Rajan Shrestha, Manita Pyakurel, Madan Prasad Upadhyay

**Affiliations:** 1 Academic & Research Department, Hospital for Children, Eye, ENT, & Rehabilitation Services, Bhaktapur, Nepal; 2 Department of Public Health, Aarhus University, Aarhus C., Denmark; 3 B.P. Eye Foundation, Kathmandu, Nepal

**Keywords:** Blindness, Malnutrition, Nepal, Prevalence, School, AOR, Adjusted odds ratio, BAZ, BMI-for-age z-score, BMI, Body mass index, CI, Confidence interval, cm, Centimetre, COR, Crude odds ratio, HAZ, Height-for-age z-score, kg, Kilogram, REF, Reference category, SD, Standard deviation, WAZ, Weight-for-age z-score, WHO, World Health Organization

## Abstract

Cross-cutting issues like nutrition have not been adequately addressed for children with severe visual impairment studying in integrated schools of Nepal. To support advocacy, this study aimed to determine the nutritional status of this vulnerable group, using a descriptive cross-sectional design involving 101 students aged 5–19 years from two integrated public schools near Kathmandu Valley and two in western Nepal. The weight-for-age z-score (WAZ), height-for-age z-score (HAZ), and body mass index-for-age z-score (BAZ) were computed and categorised using World Health Organization cut-off values (overnutrition: z-score > +2.0 standard deviations (SD), healthy weight: z-score −2.0SD to +2.0SD, moderate undernutrition: z-score ≥ −3.0SD to <−2.0SD, severe undernutrition: z-score < −3.0 SD) to assess nutritional status. A child was considered to have undernutrition for any z-scores <−2.0SD. Multivariate logistic regression was used to analyse variables linked to undernutrition. The mean age of participants was 11.86 ± 3.66 years, and the male-to-female ratio was nearly 2:1. Among the participants, 71.29% had blindness, and 28.71% had low vision. The mean BAZ and HAZ scores decreased with age. The WAZ, HAZ, and BAZ scores indicated that 6.46% were underweight, 20.79% were stunted, and 5.94% were thin, respectively. Overall, 23.76% of students had undernutrition and 7.92% had overnutrition. More than three in ten students had malnutrition and stunting was found to be prevalent. Older students and females were more likely to have undernutrition. These findings highlight the need for nutrition interventions within inclusive education settings, particularly targeting girls with visual impairments who may face compounded vulnerabilities.

Nutrition is crucial for a child’s health and development. However, the triple burden of malnutrition — undernutrition, hidden hunger, and overweight — threatens children’s survival, growth, and development, undermining the capacity of millions of children to grow and develop to their full potential.^(1)^ Disability and malnutrition could be causes and consequences of each other and are issues of public health importance.^(1–3)^ Good nutrition is also vital to those with a disability. Children with disabilities suffer poorer health outcomes, missing or delayed developmental milestones, avoidable secondary impairments, and, in extreme circumstances, premature death.^(4)^

Globally, it is estimated that in 2022, 149 million children under five years of age were stunted, 45 million were wasted, and 37 million were overweight. Among children and adolescents aged 5–19 years, 390 million were overweight and 190 million were thin.^(5)^ In Nepal, the national prevalences of stunting, wasting, and underweight for under-five years children stand at 25%, 8%, and 19%, respectively.^(6)^ Among community school students, 27.5% were underweight, 23% were stunted, and 16.6% had thinness.^(7)^ Children with disabilities are at higher risk of becoming malnourished than their non-disabled counterparts.^(8)^ It starts at the family level with feeding practices like continuing a liquid-only diet and believing the child cannot take solid foods.^(2)^ Some societies encourage mothers not to breastfeed, deny, or provide less nutritious food to their disabled children than their non-disabled siblings.^(9)^ Parents, caretakers, and service providers of children with disabilities may lack knowledge of feeding them effectively, teaching the child to feed itself, or following culturally determined gender preferences to prioritise the nutritional needs of a disabled boy over that of a disabled girl.^(2)^ The dietary needs of children with disabilities are scarcely addressed or are not included in nutritional needs at the community or population level.^(4,9)^

A systematic review showed that children with disabilities were almost thrice more likely to be underweight and nearly twice as likely to experience stunting and wasting than controls.^(10)^ A study from special schools, daycare centres, and rehabilitation homes in the Kathmandu Valley of Nepal showed that among 345 children with disabilities, 62.6% were malnourished. Among them, 44.6% were stunted, 33.3% were underweight, 19.4% were thin, and 12.2% were overweight.^(11)^ Among children with disabilities, children with visual impairment are more prone to malnutrition.^(12)^ They often have difficulty learning to feed themselves than children with adequate vision and are often perceived as ‘poor eaters’.^(13)^ Studies in Europe have shown that such children are overweight or obese due to imbalanced diets^(14)^ or physical fitness.^(15)^ The global prevalence of blindness among children is 0.17%.^(16)^ In Nepal, the prevalence of blindness and low vision among children has been reported as high as 0.06% and 0.5%, respectively.^(17)^ Heredity and unknown aetiology contribute to more than two-thirds of childhood blindness in Nepal.^(18)^

Nepal has developed and implemented different policies and strategies to ensure children with severe visual impairments learn in inclusive settings in Nepal.^(19,20)^ However, cross-cutting issues like nutrition have not been sufficiently addressed for children with severe visual impairment. There is a dire need for evidence about the nutritional status of children with severe visual impairment in Nepal to support advocacy efforts. Hence, we aimed to assess the prevalence of malnutrition among children with severe visual impairment studying in integrated schools for children with blindness and vision impairment in Nepal.

## Methods

### Study design and setting

This quantitative descriptive cross-sectional study was conducted in 4 different integrated public schools in Nepal from September to November 2021. Based on the higher number of children with severe visual impairment enrolled, we purposively selected four schools from four different districts: Banke, Kathmandu, Lalitpur, and Surkhet. We reviewed the data available from the Inclusive Education Section Center of Education and Human Resource Development, Nepal for the selection of integrated schools. Two schools from Kathmandu and Lalitpur were in the Kathmandu Valley, near the capital city, Kathmandu, and two from Banke and Surkhet were from western Nepal. These four integrated schools provide inclusive education for children with blindness and low vision. Inclusive education is a strategy that identifies children who, for any reason, are excluded from mainstream education, such as children with blindness and vision impairment, and promotes a process of social and academic inclusion for all children within the school.^(21)^ Students with vision impairment at these schools live in hostel settings with shared meals.

### Study population

The study population consisted of students with severe visual impairment. For the recruitment, we conducted detailed ocular examinations with the help of consultant Pediatric ophthalmologists and optometrists to determine the visual impairment status. Severe visual impairment in this study was grouped into children with blindness or low vision. A child was considered to have blindness if her/his eye had visual acuity of less than 3/60 in the better eye with the best possible correction.^(22)^ A child was considered to have low vision if her/his visual acuity was less than 6/18 but equal to or better than 3/60 with the best possible correction.^(22)^

### Sample size and sampling

The required sample size was calculated using the single population proportion formula:

n = (Z^2^ × p × (1 - p))/d²

where,

Z = 1.96 at 95% confidence level,

p = 73%^(12)^

d = 9% margin of error

The initial calculated sample size was 94.

With 5% non-response rate, the final minimum sample size for the study was 99 participants.

There were 112 students diagnosed with blindness or low vision. We conveniently enrolled all children with severe visual impairment studying in the selected schools to participate in this study. We excluded students with incomplete birthdate records and those with other known disabilities from the study. Among 112, we included 101 students in this study as one student also had a physical disability, and the birthday records of 10 students were incomplete in the school records.

### Study tools

We developed a semi-structured questionnaire based on literature review and experts’ advice. Our team pre-tested the draft questionnaire among ten children with blindness and low vision from an integrated school in Bhaktapur.

### Data collection

Four enumerators (two males and two females) with a background in public health were trained for one day to ensure uniformity in data collection methods. They were instructed to operate the digital weighing scale and a portable stadiometer to measure weight and height, respectively. Male enumerators measured boys, and female enumerators measured girls to maintain gender appropriateness. They were also trained to approach potential participants and explain the purpose of the study. The weighing scale was calibrated to zero before each new measurement. During the measurements, children removed their shoes and any bulky outer clothing. Weight was recorded to the nearest 0.1 kilogram (kg). For height measurement, children stood upright with their heels touching the base of the stadiometer, their shoes removed, and their backs straight. Height was recorded to the nearest 0.1 centimetre (cm). Each student’s weight and height were measured three times, and the average of the three measurements was documented. The enumerators also reviewed the school records for date of birth of each student.

### Outcome variables

The weight-for-age z-score (WAZ), height-for-age z-score (HAZ), and BMI-for-age z-score (BAZ) were used to measure the nutritional status of children. The z-scores were categorised using the World Health Organization’s (WHO) cut-off values (overnutrition: z score > +2 SD, healthy weight: z score −2.0 to +2.0 SD, moderate undernutrition: z score ≥ −3.0 to <−2.0 SD, severe undernutrition: z score < −3.0 SD) to determine the nutritional status of children. A child was considered underweight if WAZ < −2.0 SD, stunted if HAZ < −2.0 SD, and thin if BAZ < −2.0 SD. A child was considered to have undernutrition if he/she was either underweight or, stunted or thin.

### Data analysis

We entered data in Microsoft Excel 2019 (Microsoft Inc., Redmond, Washington, US) and analysed using WHO Anthro+ software 3.2.2 version (World Health Organization, Avenue Appia, Geneva, Switzerland) and IBM Statistical Package for Social Sciences 26.0 version (IBM Inc., Armonk, New York, US). The frequencies and percentages were computed to describe the sample and related variables. The chi-square test of association was used to determine the relationship between the outcome variable and each independent variable. The odds ratio and a 95% confidence level were estimated to identify the factors associated with undernutrition. All variables were included in a multivariate logistic regression analysis to determine the effect adjusted for each variable. The statistical significance level was declared at a p-value less than 0.05.

## Results

Of the 112 children with blindness and low vision, 101 were included in the study. Among the 11 children excluded from the study, one had a physical disability, and the birthday records of 10 students were incomplete in the school records.

The mean age of the study participants was 11.86 ± 3.66 years, and the male-to-female ratio was nearly 2:1. Nearly one-fourth of the study participants were from western Nepal, and more than 7 in 10 (71.29%) of study participants had blindness. (Table 1)

**Table 1.** Socio-demographic characteristics of children with severe visual impairment

Characteristics	Frequency (N=101)	Percentage
Sex		
Male	67	66.34
Female	34	33.66
Age		
5–9 years	31	30.70
10–14 years	40	39.60
15–19 years	30	29.70
Site		
Inside Kathmandu Valley	75	74.26
Western Nepal	26	25.74
Vision Impairment		
Blindness	72	71.29
Low vision	29	28.71

The mean weight and height of study participants were 39.80 ± 14.23 kg and 141.66 ± 17.56 cm. The mean WAZ score was −0.007 ± 1.388, and the mean BAZ and HAZ scores decreased with age. This indicates that the average of children was smaller and lighter than the growth standard. (Table 2)

**Table 2.** Anthropometric measurements of children with severe visual impairment grouped by ages

Anthropometric measurements	5–9 years (N=31)	10–14 years (N=40)	15–19 years (N=30)	Total (N=101)
Weight (mean ± SD) kg	25.797 ± 7.095	42.340 ± 12.578	50.893 ± 9.334	39.803 ± 14.225
Height (mean ± SD) cm	122.009 ± 9.763	147.162 ± 13.039	154.613 ± 10.385	141.655 ± 17.559
BMI (mean ± SD)) kg/m^2^	17.003 ± 2.351	19.180 ± 4.029	21.400 ± 4.201	19.171 ± 4.007
BMI for age z-score (mean ± SD)	0.535 ± 1.191	0.034 ± 1.433	−0.061 ± 1.407	0.160 ± 1.366
Height for age z-score (mean ± SD)	−0.651 ± 1.277	−1.054 ± 1.566	−2.002 ± 1.173	−1.212 ± 1.463
Weight for age z-score (mean ± SD)	−0.007 ± 1.388			−0.007 ± 1.388

BMI: Body mass index; cm: Centimetre; kg: Kilogram; m: Metre; SD: Standard deviation.

The WAZ scores showed that the same number (6.46%) of children were underweight and overweight for their age. According to HAZ scores, 20.79% of children were stunted. The BAZ scores showed that 5.94% of children were thin for their age. Overall, 23.76% of children had undernutrition, and 8 (7.92%) had overnutrition. Among children with blindness, 22.22% had undernutrition; among children with low vision, an even higher proportion (27.59%) had undernutrition. (Table 3)

**Table 3.** Nutritional status of children with severe visual impairment

	Visual impairment		Age Category			Gender		Site	
Nutrition category	Blindness (%)	Low vision (%)	5–9 years (%)	10–14 years (%)	15–19 years (%)	Male (%)	Female (%)	Kathmandu Valley (%)	Western Nepal (%)
Weight for Age	N=27	N=4	N=31			N=24	N=7	N=28	N=3
Severe undernutrition	1 (3.70)	0 (0.00)	1 (3.23)			1 (4.17)	0 (0.00)	0 (0.00)	1 (33.33)
Moderate undernutrition	1 (3.70)	0 (0.00)	1 (3.23)			1 (4.17)	0 (0.00)	1 (3.57)	0 (0.00)
Optimal Nutrition	23 (85.20)	4 (100.00)	27 (87.08)			21 (87.49)	6 (85.71)	25 (92.59)	2 (66.67)
Overnutrition	2 (7.40)	0 (0.00)	2 (6.46)			1 (4.17)	1 (14.29)	2 (7.14)	0 (0.00)
Height for Age	N=72	N=29	N=31	N=40	N=30	N=67	N=34	N=75	N=26
Severe undernutrition	7 (9.72)	4 (13.79)	1 (3.23)	5 (12.50)	5 (16.67)	4 (5.97)	7 (20.59)	5 (6.67)	6 (23.08)
Moderate undernutrition	8 (11.11)	2 (6.90)	1 (3.23)	3 (7.50)	6 (20.00)	8 (11.94)	2 (5.88)	8 (10.67)	2 (7.69)
Optimal Nutrition	56 (77.78)	23 (79.31)	28 (90.31)	32 (80.00)	19 (63.33)	54 (80.60)	25 (73.53)	61 (81.33)	18 (69.23)
Overnutrition	1 (1.39)	0 (0.00)	1 (3.23)	0 (0.00)	0 (0.00)	1 (1.49)	0 (0.00)	1 (1.33)	0 (0.00)
BMI for Age	N=72	N=29	N=31	N=40	N=30	N=67	N=34	N=75	N=26
Severe undernutrition	0 (0.00)	1 (3.45)	0 (0.00)	0 (0.00)	1 (3.33)	1 (1.49)	0 (0.00)	0 (0.00)	1 (3.85)
Moderate undernutrition	3 (4.17)	2 (6.90)	2 (6.45)	2 (5.00)	1 (3.33)	3 (4.48)	2 (5.88)	1 (1.33)	4 (15.38)
Optimal Nutrition	61 (84.72)	24 (82.75)	26 (83.87)	33 (82.50)	26 (86.67)	54 (80.60)	31 (91.18)	64 (85.34)	21 (80.77)
Overnutrition	8 (11.11)	2 (6.90)	3 (9.68)	5 (12.50)	2 (6.67)	9 (13.43)	1 (2.94)	10 (13.33)	0 (0.00)
Nutritional status	N=72	N=29	N=31	N=40	N=30	N=67	N=34	N=75	N=26
Undernutrition	16 (22.22)	8 (27.59)	3 (9.68)	8 (20.00)	13 (43.33)	14 (20.90)	10 (29.41)	14 (18.67)	10 (38.46)
Optimal Nutrition	49 (68.06)	20 (68.96)	25 (80.64)	27 (67.50)	17 (56.67)	47 (70.15)	22 (64.71)	53 (70.66)	16 (61.54)
Overnutrition	7 (9.72)	1 (3.45)	3 (9.68)	5 (12.50)	0 (0.00)	6 (8.95)	2 (5.88)	8 (10.67)	0 (0.00)

BMI: Body mass index.

Females, older age, children from western Nepal, and children with low vision were more likely to have undernutrition. The multivariate analysis showed that age groups were significantly associated with undernutrition among children. (Table 4)

**Table 4.** Factors associated with undernutrition among children with severe visual impairment

	Undernutrition					
Variables	Yes (%)	No (%)	COR (95% CI)	p-value	AOR (95% CI)	p-value
Sex						
Male	14 (20.90)	53 (79.10)	REF		REF	
Female	10 (29.41)	24 (70.59)	1.577 (0.614–4.054)	0.34	1.374 (0.499–3.784)	0.54
Age						
5–14 years	11 (15.49)	60 (84.51)	REF		REF	
15–19 years	13 (43.33)	17 (56.67)	4.171 (1.586–10.968)	0.003*	3.697 (1.334–10.247)	0.01*
Sites						
Inside Kathmandu Valley	14 (18.67)	61 (81.33)	REF		REF	
Western Nepal	10 (38.46)	16 (61.54)	2.723 (1.021–7.260)	0.04*	2.222 (0.710−6.953)	0.17
Vision Impairment						
Blindness	16 (22.22)	56 (77.78)	REF		REF	
Low vision	8 (27.59)	21 (72.41)	1.333 0.498–3.573)	0.57	1.456 (0.444–4.772)	0.54

AOR: Adjusted odds ratio, CI: Confidence interval; COR: Crude odds ratio, REF: Reference category, *: Statistically significant at p<0.05.

## Discussions

In the study of children with blindness and low vision in integrated schools, 23.76% were underweight, stunted, or thin for their age. The mean BAZ and HAZ scores decreased with age.

The proportion of undernutrition among students with visual impairment in our study is comparable to that of other school-going students in Nepal. Similar rates of children with stunting were found in 9–17 year old students from public and private schools of Pokhara (16.7%),^(23)^ and 4–16 year old students from rural Kavrepalanchowk (24.5%).^(24)^ Another study covering similar-aged students from 98 schools with a mid-day meal programme across 44 municipalities in all seven provinces of Nepal also showed that 23.0% were stunted.^(7)^ Addressing childhood and adolescent malnutrition has been a priority for the government of Nepal, and various initiatives have been launched over the past 50 years to improve their nutritional status.^(25)^ However, undernutrition, and particularly stunting remains a persistent issue among under-five children, those in middle childhood, and adolescents in Nepal.^(6,7,23,26)^ This highlights the need for broader efforts to combat malnutrition and address the nutritional needs of all students, ensuring that children with visual impairment are not overlooked in these interventions.

The proportion of children with undernutrition was nearly thrice that of children who were overweight in our study. In contrast, almost all studies done among children with vision impairment in European countries reported a higher proportion of overweight (24–36.2%)^(14,15,27)^ than undernutrition (9.5%).^(28)^ This may be due to differences in institutional care for such children; lack of awareness, dietary habits, and access to food resources could have contributed to higher rates of undernutrition in our study settings. Among children with blindness (excluding low vision), 7.40% were underweight, 20.83% were stunted, and 9.72% were overweight in our study. A study among children with blindness aged 5–19 years in Mosul, Iraq, showed that 15% were underweight, 35% were stunted and 45% were overweight.^(29)^ An earlier study from Lebanon among children with blindness showed that 16.35% were underweight and 18.85% were stunted.^(30)^ All these results suggest that malnutrition is a major concern among children with severe visual impairments in low and middle-income countries, and we are struggling to cater to their needs. However, national and international nutritional guidelines lack specific guidance for this vulnerable group.^(25,31)^ Tailored guidance is essential to ensure these children receive adequate nutrition to support their growth, development, and overall well-being.

The female students with severe visual impairment were more likely to have undernutrition than males in our study, though the association was not statistically significant. The prevalence of stunting was higher among females than males in our study. This could be due to gender-based discrimination, cultural norms, and limited resources. A study among children with disabilities in the Kathmandu Valley of Nepal showed that females were more likely to be stunted than males.^(11)^ In a study among children with cerebral palsy in Turkey, females were more likely to be underweight than males.^(32)^ Among apparently healthy school adolescents in India, undernutrition was also higher among females than males.^(33,34)^ Children with disability receive less nutritious food than their non-disabled siblings,^(9)^ and poor nutrition in Nepal has been linked to gender inequality, with girl children at risk from early childhood,^(35)^ making female children with severe visual impairments more susceptible to poor nutrition. Boys are often viewed as having greater food needs, while girls are expected to eat less and focus on domestic work. Cultural norms also limit their eating habits and behaviours.^(36)^ In addition, stunted females tend to have stunted offspring, creating an intergenerational cycle of poverty.^(37)^ Hence, health workers, parents, and caregivers need to give special attention to females by providing nutritious meals to meet their dietary requirements, break this cycle, and ensure the well-being of both current and future generations.

The undernutrition increased with age in our study. Stunting was the major reason for undernutrition in our study, and it increased with age. A study among apparently healthy adolescents in West Bengal, India, also showed that with an increase in age, the adolescent showed a significantly higher prevalence of stunting, and 17-year-old adolescents had approximately 4 times higher risk of being stunted than 10-year-old adolescents.^(34)^ Stunting results from chronic undernutrition and typically manifests in later childhood.^(11)^ Children with severe visual impairments are either discriminated against during feeding,^(9)^ or their caretakers or parents are unaware of their nutritional needs. Caregivers and parents often lack the knowledge and skills to provide balanced and nutritious meals that meet such children’s dietary requirements. Hence, studies have suggested that supporting parents to meet their children’s developmental needs^(38)^ and boosting caregivers’ confidence can foster independence during mealtimes and help establish healthy eating habits for visually impaired children.^(39)^ In communities like Nepal, where short stature is common, stunting is frequently overlooked, emphasising the necessity of measuring length/height alongside weight for nutritional assessment of children.^(40)^ Early identification and intervention are crucial in addressing malnutrition among children with severe visual impairment. School teachers, school nurses, and caregivers can play pivotal roles in recognising signs of malnutrition, like poor eating habits. Early rehabilitation is essential for children with poor nutrition to enhance their nutritional health and overall well-being.

The children studying in integrated schools in western Nepal were more likely to have undernutrition than students from Kathmandu Valley. Childhood undernutrition among under-five children also shows that the central region, where Kathmandu Valley is a part, has a lower prevalence of overall undernutrition than western Nepal.^(41)^ Though all the students were in residential schools, the difference might be due to access to the quality and quantity of food provided to students in Kathmandu Valley. Kathmandu Valley, being the capital region, the schools have better access to resources, health services, and nutrition programmes as compared to western Nepal. The western region of Nepal has long struggled with food crises, and food insecurity has further compounded the challenges of addressing malnutrition in the area.^(42,43)^

The study’s strength lies in its focus on the nutritional status of children with severe visual impairment, a group often overlooked in research. However, there are some limitations. The study did not include out-of-school children. The use of convenience sampling from four purposively selected schools limits the generalizability of the findings. Another limitation is the small sample size, which restricts the ability to conduct subgroup analyses. Additionally, the study was limited to quantitative methods, which constrains the understanding of contextual and behavioural factors influencing nutritional outcomes. This study groups underweight, stunting, and thinness under the broad term ‘undernutrition’, which may oversimplify their distinct physiological, temporal, aetiological, and developmental characteristics. While thinness typically reflects acute malnutrition, stunting indicates chronic nutritional deprivation, and underweight may result from either or both. These conditions also differ in their underlying causes, health risks, and relevance across age groups. Aggregating them into a single category may limit the study’s ability to capture the complexity of nutritional challenges among children with visual impairments.

Future research could compare the nutritional status of students with visual impairments to that of their peers in the same school setting, to identify disparities within shared environments. To elucidate the determinants of malnutrition in this vulnerable population, future studies should examine factors such as parental education, maternal and sibling nutrition, economic influences like food security, and behavioural aspects, including health-seeking behaviour, dietary diversity and feeding practices. Using qualitative methods, future research could also incorporate the perspectives of children with visual impairments, their parents, peers, caretakers, and teachers, providing valuable insights into the contextual and behavioural factors influencing nutritional outcomes. Furthermore, with larger sample size, future investigations could explore the distinct factors contributing to different forms of malnutrition separately, like stunting, underweight and thinness, as these reflect varying socio-cultural, economic, physiological, and temporal dimensions. A longitudinal or case-control approach would facilitate the examination of causality and the long-term effects of nutritional status on the health and development of children with visual impairments. These studies should also incorporate randomised, larger, and more diverse samples drawn from various ecological zones, as well as rural and urban settings, out-of-school populations, and socio-cultural contexts across Nepal, to enhance the generalizability of the findings.

Nearly three in ten children with severe visual impairment were found to have malnutrition and girls disproportionately affected by undernutrition. Our findings highlight the need for nutrition interventions within inclusive education settings in integrated schools. School health programmes in integrated schools should include targeted nutrition screening and interventions for children with disabilities, ensuring they receive regular assessments, fortified meals, and supplements as needed. This is particularly important for girls with visual impairments who may face compounded vulnerabilities. Since these children are already excluded from many opportunities, addressing their nutritional needs is critical for promoting their overall well-being, development and inclusion. Hence, at the policy level, nutrition, health, and disability-inclusive frameworks must recognise children with disabilities as a high-risk group. They should include tailored guidelines, dedicated resource allocation within strategic plans, and monitoring mechanisms to ensure their specific needs are addressed effectively.
